# Cerebral Hemodynamics, Right-to-Left Shunt and White Matter Hyperintensities in Patients with Migraine with Aura, Young Stroke Patients and Controls

**DOI:** 10.3390/ijerph19148575

**Published:** 2022-07-14

**Authors:** Nicoletta Brunelli, Claudia Altamura, Carlo A. Mallio, Gianguido Lo Vullo, Marilena Marcosano, Marcel Bach-Pages, Bruno Beomonte Zobel, Carlo Cosimo Quattrocchi, Fabrizio Vernieri

**Affiliations:** 1 Headache and Neurosonology Unit, Neurology Unit, Campus Bio-Medico University Hospital Foundation, 00128 Rome, Italy; c.altamura@policlinicocampus.it (C.A.); marilena.marcosano@unicampus.it (M.M.); f.vernieri@policlinicocampus.it (F.V.); 2 Radiology Unit, Campus Bio-Medico University Hospital Foundation, 00128 Rome, Italy; c.mallio@policlinicocampus.it (C.A.M.); g.lovullo@policlinicocampus.it (G.L.V.); b.zobel@policlinicocampus.it (B.B.Z.); c.quattrocchi@policlinicocampus.it (C.C.Q.); 3 Department of Plant Sciences, University of Oxford, Oxford OX1 3RB, UK; m.bach.pages@gmail.com; 4 FENIX Group International, LLC, Reading, PA 19601, USA

**Keywords:** cerebral hemodynamics, right-to-left shunt, white matter hyperintensities

## Abstract

Background: Migraine with aura (MA) patients present an increased risk of cerebrovascular events. However, whether these patients present an increased white matter hyperintensities (WMHs) load compared to the general population is still under debate. Our study aimed to evaluate the relationship between cerebral hemodynamics, right-to-left shunt (RLS) and WMHs in MA patients, young patients with cryptogenic stroke or motor transient ischemic attack (TIA) and controls. Methods: We enrolled 30 MA patients, 20 young (<60 years) patients with cryptogenic stroke/motor TIA, and 10 controls. All the subjects underwent a transcranial Doppler bubble test to detect RLS and cerebral hemodynamics assessed by the breath holding index (BHI) for the middle (MCA) and posterior (PCA) cerebral arteries. Vascular risk factors were collected. The WMHs load on FLAIR MRI sequences was quantitatively assessed. Results: The stroke/TIA patients presented a higher prevalence of RLS (100%) compared with the other groups (*p* < 0.001). The MA patients presented a higher BHI compared with the other groups in the PCA (*p* = 0.010) and higher RLS prevalence (60%) than controls (30%) (*p* < 0.001). The WMHs load did not differ across groups. BHI and RLS were not correlated to the WMHs load in the groups. Conclusions: A preserved or more reactive cerebral hemodynamics and the presence of a RLS are likely not involved in the genesis of WMHs in MA patients. A higher BHI may counteract the risk related to their higher prevalence of RLS. These results need to be confirmed by further studies to be able to effectively identify the protective role of cerebral hemodynamics in the increased RLS frequency in MA patients.

## 1. Introduction

Migraine is a syndrome of the central nervous system widespread among the general population [1,2] that leads to high disability and social impacts [3]. Even though of predominantly neurological origin, it is comorbid with a plethora of medical conditions affecting other somatic systems [4]. Migraine patients have an increased long-term risk of cardio and cerebrovascular events [5], with a further increase in relative risk in migraine with aura (MA; 1.56–2.41) compared with migraine without aura (MO; 1.11–1.83) patients [6].

The physio-pathological link between stroke and migraine has been hypothesized as the effect of different etiopathogenesis: from thromboembolism to hemodynamic dysfunction and energetic failure [7]. Other mechanisms such as cortical spreading depression (CSD), biochemical alterations, arterial vasospasm, cerebral edema, and platelet aggregation have also been proposed [8]. Subclinical atherosclerosis, such as the intima media thickening, could be a marker of endothelial dysfunction linking vascular disease to migraine [9,10,11,12]. Some substances such as nitric oxide, endothelin, von Willebrand factor and platelet-activating factor that are released by the endothelium in reaction to changes in the local environment can lead to local inflammation and thrombosis. This phenomenon, recognized as endothelial activation [13], predisposes patients with migraine to vascular diseases. A pro-inflammatory and pro-coagulative state has been observed in migraine patients, especially in MA, chronic migraine (CM) and women, mostly those in the premenopausal period [14,15,16]. The most significant factor associated with risk of stroke in migraine patients is the high estrogen state, especially in MA patients with a smoking habit [17]. Platelet activation could explain the increased vascular risk in migraine patients [18,19], supporting the evidence that antiplatelet therapy can relieve MA [20] also in patients without right-to-left shunt (RLS) [21]. It is not clear whether thrombophilia can be observed in migraine patients: some studies report an increased prevalence of prothrombotic polymorphisms [22,23], but no univocal results are available [24]. A transient hypoperfusion caused by the pro-thrombotic and pro-inflammatory states has been hypothesized as the origin of migraine attack [25]. This scenario offers the possibility to detect micro-emboli in the cerebral circulation in MA patients with a high prevalence of RLS [26]. In the Oxford Vascular study cohort, migraine was associated with cryptogenic stroke and transient ischemic attack (TIA), suggesting a causative role for migraine or a shared etiopathogenesis between them, and may be attributed to the presence of RLS [27]. Among other mechanisms, and besides the presence of a high prevalence of RLS in MA patients, it has been proposed that an impairment of cerebral hemodynamics subtends the pathological link between MA and cerebrovascular disease.

Vasomotor reactivity (VMR) can be accurately and noninvasively investigated by transcranial Doppler (TCD) [28] and reflects the intracranial arterioles’ potential to dilate in response to hypercapnia. Hence, VMR is known to be a marker of efficiency and health of cerebral circulation. VMR correlates with stroke risk [29], and preserved VMR is also associated with better functional outcomes following stroke [30]. Impaired VMR represents a negative prognostic factor for vascular and degenerative cognitive deterioration [31,32].VMR and neurovascular coupling are impaired during migraine attacks, especially during the aura of MA [33,34]. On the contrary, in the interictal period, most studies reported a preserved or higher VMR in migraine patients, especially in MA patients, when compared with controls [35,36,37,38,39,40,41,42]. However, some studies report impaired VMR in the interictal period, mainly in posterior circulation [43,44,45]. Moreover, VMR seems to be lower in CM and in MA patients taking estrogens [21].

The migraine brain seems to also be predisposed to vascular insults due to the excessive energy requirement and inefficient use during the transient or persistent sensory hypersensitivity. Among the patients suffering stroke, those who also suffer migraine and especially MA, show a decreased ratio between infarcted and hypo-perfused tissue [46]. Furthermore, migraine patients suffer cortical infarcts more often [47]. Therefore, it is known that MA patients present an increased stroke risk. However, whether MA patients present an increased White Matter Hyperintensities (WMHs) load compared with the general population is still under debate. In the CAMERA-2 (Cerebral Abnormalities in Migraine, an Epidemiological Risk Analysis-2) study, WMHs load was associated with migraine prevalence and progression rate [48]. In a longitudinal MRI study in migraine patients, clinically silent brain WMHs were found at 3 years. However, the absence of a control group in this study precluded a definitive conclusion about the nature of these changes [49]. Other studies also reported an increased prevalence of WMHs and silent posterior circulation territory infarcts in migraineurs [50,51]. The pathogenesis of the lesion development is not completely known. Several attack-related factors have been considered in the formation of WMHs such as the disease duration and the attack frequency [52,53]. Among these factors, the effects of an impaired Cerebral Hemodynamics and the presence of RLS could at least partly account for the increased WMHs risk. A recent study found migraineurs had lower VMR than controls and migraineurs with RLS had lower VMR than those without RLS. In the same study, migraineurs with WMHs had lower VMR than those without WMHs [54]. Other authors have found a migraine-specific association between a reduced VMR and an increased number of WMHs in healthy nonelderly patients with migraine [55] but to date, there are no univocal results.

The hypothesis underlying the study is that a preserved or improved VMR in MA patients could have a protective role in the WMHs genesis.

Our study aimed to evaluate the relationship between cerebral hemodynamics, RLS and WMHs in patients affected by MA, young patients with cryptogenic stroke or motor transient ischemic attack and controls.

## 2. Materials and Methods

### 2.1. Data Collection

We consecutively enrolled 30 MA patients (mean age 35.2 [10.9] years), 20 young (<60 years) patients with cryptogenic stroke or motor TIA (mean age 39.1 [7.4] years) and 10 controls age-matched to the MA patients (mean age 40.5 [8.3] years). We included only motor TIA as aphasic/sensory TIA may resemble episodes of typical aura without headache.

The individuals were enrolled from among subjects referred to our neurosonology lab to undergo transcranial Doppler (TCD) examinations and bubble tests to detect RLS presence. We classified MA according to the International Classification of Headache Disorders [56]. We defined cryptogenic stroke/motor TIA according to the TOAST (Trial of Org 10,172 in Acute Stroke Treatment) classification. We used continuous wave Doppler and color flow B-mode Doppler ultrasound (Philips iU22, Bothell, WA, USA) to assess extra- and intra-cranial arteries. For all participants, the exclusion criteria included: malignancy, other neurological disorders, cerebral or neck vessels steno-occlusive diseases, ongoing vasoactive therapy (calcium antagonist, beta-blockers) or other migraine prophylactic treatments. We collected vascular risk factors through medical history interviews with all subjects. We defined hypertension as a history of high blood pressure, a systolic blood pressure ≥ 140 mmHg, diastolic blood pressure ≥ 90 mmHg, or the use of an antihypertensive. Diabetes was defined as a fasting blood glucose level of ≥126 mg/dL or current treatment for diabetes [57]. Smoking was defined as active tobacco smoking. The active use of estrogenic-progestin therapy was also collected [7].

Additionally, for the MA patients, we also collected the following information: MA family history, aura duration, frequency of MA attacks in the previous year and aura type (visual, sensitive, aphasic, and complex).

### 2.2. Hemodynamic Evaluation

**Figure 1:**
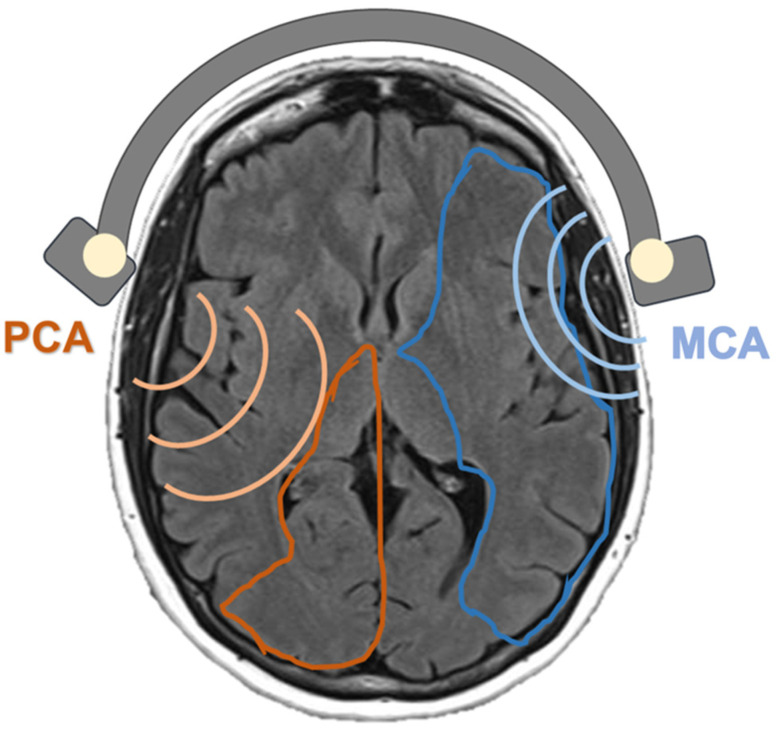
Diagram of the MCA/PCA insonation.

TCD examinations were performed according to the guidelines of our neurosonology lab [58]. To evaluate VMR, we performed a breath-holding (BH) test. Middle (MCA) and posterior (PCA) cerebral arteries were continually and concomitantly insonated at rest and during an apnea of at least 30 s in all individuals. The breath-holding index (BHI) was calculated as the percent increase in cerebral mean blood flow velocity (MBFv) after the apnea test with respect to baseline and corrected by the length of apnea. A neurosonologist blind to the subjects’ conditions computed the BHIs off-line. The VMR evaluation preceded TCD with a microbubble test in all participants. The BH test was performed within 7 days from symptoms onset in all subjects (at least within 48 h in patients with stroke) and always within the interictal period in patients with MA. In the stroke patients, MCA and PCA were insonated in the non-affected side, while in the MA patients and controls, MCA was insonated on the left side and PCA on the right (Figure 1).

### 2.3. Transcranial Doppler Bubble Test

To perform the TCD bubble test, two ultrasound transducers held by a headband were placed on the temporal bone to insonate MCAs bilaterally (Multidop-X-DWL; Elektronische-Systeme GmbH, Sipplingen, Germany). We invited patients to assume a semi-sitting position and to perform a Valsalva maneuver (VM) of 10 s to obtain a reduction of 25% in peak systolic velocity before the test. An 18-gauge needle was positioned at a 45° angle into the cubital vein with the arm straight; 9 mL of saline solution was mixed with 1 mL of air and 1 mL of autologous blood to obtain microbubbles. We injected the microbubbles mix as a bolus at rest and after the VM. Cerebral emboli were detected as typical high-intensity transient signals (HITS) within TCD spectral curves. To quantify the HITS, the recorded TCD spectra were analyzed offline by an expert neurosonologist blind to the subjects’ clinical conditions.

To obtain a unique score in order to stratify RLS severity at rest and after VM, the RLS grading system was redefined [58], merging the HITS counts in the two conditions. RLS was classified for severity according to the following criteria: 0 = 0 HITS detected at rest or after VM; 1 = 1–10 HITS/side at rest and/or <20/side after VM; 2 = 10–20/side at rest and/or “shower” effect after VM; 3 = “shower” effect at rest or “curtain” effect after VM; and 4 = “curtain” effect at rest. We defined severe RLS as grade 3 (i.e., “shower” effect at rest or “curtain” effect after VM) according to Elgendy et al. [59]. HITS found after ≥30 s from microbubbles injections were excluded, reflecting lung arterio-venous shunts [21].

### 2.4. MRI Acquisition and WMHs Quantification

A 1.5 Tesla MRI system (Magnetom Avanto B13, Siemens, Erlangen, Germany) configured with a 12-element head matrix coil was used to acquire the images. The brain MRI protocol included the axial FLAIR sequence (repetition time [TR], 8000 ms; echo time [TE], 102 ms; inversion time, 3650 ms; matrix, 256 × 256; field of view [FOV], 26 × 30 cm; slice thickness 3 mm) [60]. WMHs were defined as focal, confluent or diffuse hyperintense lesions on axial FLAIR images. Measurements of brain WMHs volume were obtained as follows. First, the regions of interest (ROIs) of the WMHs area (cm^2^) were segmented and calculated for each subject using a function of OsiriX MD v.2.6 software. The values of WMHs area were collected separately according to the vascular territories of the MCA and PCA. Then, WMHs area values were multiplied by the thickness of the image slice in order to obtain values of WMHs volume (cm^3^) for each subject. Two experienced neuroradiologists (C.A.M., 10 years of experience; G.L.V., 5 years of experience) visually inspected all the segmentations and verified by consensus the accuracy of the measurements [61].

### 2.5. Statistical Analysis

This is a preliminary study on a convenience sample. Statistical analyses were performed using SPSS version 26.0 (SPSS Inc., Chicago, IL, USA). Interval variables are expressed as means with standard deviation (SD) or medians with interquartile range (IQR) according to data distribution assessed with the Kolmogorov–Smirnov test. Frequencies were analyzed with χ^2^ test. The Kruskal–Wallis test was used to compare non-normal variables across groups, and the Mann–Whitney test for between groups. Normal variables were tested using ANOVA. Stepwise linear regression was applied to assess the correlation between BHI (independent variable) and WMHs volume (dependent variable) or number (dependent variable), taking into account diagnosis and RLS (independent variable). The statistical significance was set at *p* < 0.05.

## 3. Results

We consecutively enrolled 30 MA patients, 20 young (defined as <60 years) patients with cryptogenic stroke/motor TIA and 10 controls age-matched with the MA patients. The aura characteristics of the MA patients are summarized in Table 1. The demographic and clinical characteristics, vascular risk factors, hemodynamic parameters and WMHs load for each group of subjects are summarized in Table 2.

The stroke/TIA patients presented higher prevalence of RLS (100%). The MA patients presented higher BHI compared with the other groups in the PCA (*p* = 0.010). The WMHs load did not differ across groups. The MCA and PCA BHI was not correlated with WMHs load even when taking into account the diagnosis or presence of RLS.

## 4. Discussion

In this study we explored the relationship between cerebral hemodynamics, RLS and WMHs in patients affected by MA, young patients with cryptogenic stroke or motor TIA and controls. To our knowledge, this is the first study to explore WMHs simultaneously in these three different groups.

The main finding emerging from our study is that preserved or even more reactive cerebral hemodynamics and the presence of RLS are likely not involved in the genesis of WMHs in MA patients. Our results show that MA patients have preserved or higher VMR (higher BHI in the PCA) compared with the other groups. This finding is supported by previous studies reporting a preserved or higher VMR in migraine patients in the interictal period compared with controls, especially in MA patients [35,36,37,38,39,40,41,42].

Cerebral hemodynamics is a complex system able to guarantee constant brain perfusion under different conditions and depends on the orchestral action of neurogenic, myogenic, endothelial and metabolic responses. The neurogenic control is mediated by neurotransmitters with vasoactive properties that are released by sympathetic, parasympathetic and sensory neurons. The sensory neurons act by secreting calcitonin gene-related peptide (CGRP), nitric oxide (NO), serotonin, and pituitary adenylate cyclase-activating polypeptide [62]. The endothelium plays a significant role in vessel caliber regulation through the paracrine secretion of substances such as NO, adrenomedullin and endothelin 1 [16,63]. Cerebral arterioles are capable of regulating their caliber in response to systemic blood pressure (i.e., autoregulation-CA) or CO_2_/O_2_ concentration (i.e., VMR). In the presence of a biochemical trigger (i.e., CO_2_), MA patients in the interictal phase present more prominent arteriolar vasodilation in the PCA district than controls and stroke patients. This may be due to a higher release of vasoactive substances (e.g., NO) by the endothelium or to an increased sensitivity of vessel smooth muscles as hypothesized by Olesen and Thomsen [64,65]. Previous studies found a greater MCA velocity reduction induced by a CGRP infusion in migraineurs than controls, suggesting that MA patients have a vascular tree that is more sensitive to neurogenic modulation of hemodynamics [66,67]. Other mechanisms have been hypothesized on the basis of an increased sensitization in MA patients, one of which relies on CGRP release from sensory neurons. CGRP could prepare the brain to manage stressful conditions (i.e., increased metabolic demand) [68] and counterbalance the action of other substances with vasoconstrictive properties such as endothelin 1, which is released during cortical spreading depression (CSD) [69]. Therefore, CGRP-related vasodilation can be considered a vascular adaptation to the metabolic and hemodynamic consequences of CSD [70].

Our study also shows that MA patients present higher RLS prevalence (60%) than controls (30%), as already described previously [71]. The relationship between MA and RLS is a matter of unresolved debate. The increased prevalence of RLS in MA patients led to an initial enthusiasm for a vascular hypothesis of MA pathogenesis. However, this was later tempered by unsatisfactory results in intervention trials for PFO closure, which showed PFO closure did not reduce overall monthly migraine days [72,73]. We assume that the presence of RLS could allow the transit of vasoactive substances (e.g., serotonin and CO_2_) in MA patients and promote an increased BHI. In this scenario, we envisage that higher reactive cerebral hemodynamics in patients with MA may counteract the risk related to the higher prevalence of RLS and explain why WM is preserved in this population. Indeed, our results show that the WMHs load does not differ across the analyzed groups. However, whether MA patients have a higher load of WMHs is still under debate in the literature. A longitudinal MRI study in migraineurs showed clinically silent brain WMHs at 3 years [49]. Nevertheless, the lack of a control group in that study impedes the understanding of the real etiopathogenesis of these lesions. A physiological increase in age or in vascular risk factors cannot be excluded as potential causes. A recent study found an association between WMHs and the presence of vascular risk factors and between WMHs and age > 45 years in migraine patients. However, no relationship was found between WMHs and a higher frequency of attacks [74]. Another study did not find a different WMHs load between MA patients, MO patients and controls. Nevertheless, a high WMHs load was strongly associated with increasing age [75]. The same authors found that those MA patients with a high WMH load had a lower cerebral blood flow [75]. These data support the hypothesis that preserved or even more reactive cerebral hemodynamics has a protective role against WMHs. To date, only few authors have explored the role of VMR in WMHs, with one study reporting an association between reduced VMR and an increased number of WMHs in healthy non-elderly patients with migraine [55]. However, patients with migraine with aura were not included in the study. Other studies suggest that a hemodynamic dysfunction correlated with the WMHs load might play a pathogenic role in the development of vascular-related cognitive disorders [76] and in executive dysfunction and depression [77]. Therefore, TCD represents a valuable tool in the early detection, assessment, and management of patients at risk of vascular cognitive impairment and patients with MA.

Our study presents some limitations, the most relevant being the small sample size. The sample size was calculated based on previous available studies on migraine with aura and VMR [39]. The small sample size could reduce the power of the study and increase the margin of error. However, these results need to be further confirmed by larger studies. Another limitation is being a single-center study.

However, our study has some strengths. We explored the relationship between cerebral hemodynamics, RLS and WMHs in three different populations compared with each other and matched for age. We also studied how VMR and RLS could influence the presence of WMHs load, which is an innovative feature compared with previous studies.

## 5. Conclusions

In conclusion, our results support the hypothesis that preserved or even more reactive cerebral hemodynamics and the presence of a RLS are likely not involved in the genesis of WMHs in MA patients. We speculate that a higher BHI may counteract the risk related to the higher prevalence of RLS in MA patients. These results need to be confirmed by further studies to be able to effectively identify the protective role of cerebral hemodynamics in the increased RLS frequency in patients with MA. These results show TCD represents a useful instrument to assess and manage MA patients.

## Figures and Tables

**Table 1:** Aura characteristics of MA patients.

	Migraine with Aura (n = 30)
Visual Aura, n (%)	19 (63.3)
Attacks, n/year (SD)	10.4 (11.2)
Aura duration, min (SD)	40.5 (30.2)
Family history, n (%)	10 (33.3)

**Table 2:** Demographic and clinical characteristics, vascular risk factors, hemodynamic parameters and WMHs load for each group of subjects.

	Controls (n = 10)	Migraine with Aura (n = 30)	Cryptogenic Stroke/Motor TIA (n = 20)	*p*
Age, mean, (SD)	40.5 (8.3)	35.2 (10.9)	39.1 (7.4)	0.206
Sex, F, n (%)	8 (80)	26 (86.7)	11 (55)	**0.035**
Smoking Habit, n (%)	4 (40)	6 (20)	5 (25)	0.449
Estrogenic-progestinal therapy, n (%F)	0	3 (11.5)	2 (16.7)	0.460
Hypertension, n (%)	2 (20)	2 (6.7)	3 (15)	0.445
Diabetes, n (%)	0	0	0	-
Right-to-left shunt, n (%)	3 (30)	18 (60)	20 (100)	**<0.001**
Severe right-to-left shunt, n (%)	1 (10)	10 (33.3)	5 (25)	0.345
MCA basal MFV cm/s, mean (SD)	71.3 (14.1)	65.9 (11.9)	63.0 (10.0)	0.201
PCA basal MFV cm/s, mean (SD)	45.7 (15.5)	44.4 (12.4)	44.8 (11.9)	0.962
BHI MCA %/s, mean (SD)	1.56 (0.49)	1.86 (0.52)	1.59 (0.60)	0.154
BHI PCA %/s, mean (SD)	1.49 (0.38)	1.96 (0.66)	1.49 (0.51)	0.010 *
**WMHs number**				
MCA territory, median (IQr) [range]	1.6 (4.00) [0–12]	1.0 (3.0) [0–16]	2.5 (6.0) [0–16]	0.237
PCA territory, median (IQr) [range]	0 (0) [0–5]	0 (1.0) [0–2]	0 (1.0) [0–3]	0.159
Global, median (IQr) [range]	0.6 (2.25) [0–17]	1 (3.25) [0–20]	3.5 (9.5) [0–23]	0.229
**WMHs volume**				
MCA territory, median (IQr) [range]	0.016 (0.18) [0–1.4]	0.016 (0.12) [0–7]	0.09 (0.73) [0–3.7]	0.130
PCA territory, median (IQr) [range]	0 (0) [0–0.57]	0 (0.02) [0–0.6]	0 (0.08) [0–0.31]	0.093
Global, median (IQr) [range]	0.016 (0.19) [0–2]	0.039 (1.14) [0–9.6]	0.154 (0.944) [0–3.7]	0.182

F = female, MCA = middle cerebral artery, MVF = mean flow velocity, PCA = posterior cerebral artery, BHI = breath-holding index, SD = standard deviation, IQr = interquartile interval. * 0.038 (controls vs. migraine with aura patients); 0.011 (migraine with aura vs. stroke patients). Significant *p*-values are shown in bold.

## Data Availability

Anonymized data will be shared on request from any qualified investigator.
